# Laparoscopic uterine surgery as a risk factor for uterine rupture during pregnancy

**DOI:** 10.1371/journal.pone.0197307

**Published:** 2018-05-22

**Authors:** An-Shine Chao, Yao-Lung Chang, Lan-Yan Yang, Angel Chao, Wei-Yang Chang, Sheng-Yuan Su, Chin-Jung Wang

**Affiliations:** 1 Department of Obstetrics and Gynecology, Chang Gung Memorial Hospital and Chang Gung University, College of Medicine, Taoyuan, Taiwan; 2 Clinical Trial Center, Chang Gung Memorial Hospital, Taoyuan, Taiwan; Indiana University School of Medicine, UNITED STATES

## Abstract

The incidence of uterine rupture through a previous cesarean scar (CS) is declining as a result of a lower parity and fewer options for vaginal birth after cesarean. However, uterine ruptures attributable to other causes that traumatize the myometrium are on the rise. To determine whether changes in the causes of uterine rupture had occurred in recent years, we retrospective retrieved the clinical records of all singletons with uterine rupture observed in the delivery room of a Taiwanese tertiary obstetric center over a 15-year period. The overall uterine rupture rate was 3.8 per 10,000 deliveries. A total of 22 cases in 20 women (with two of them experiencing two episodes). Seven uterine ruptures occurred through a previous cesarean scar (CS ruptures, 32%), 13 through a non-cesarean scar (non-CS ruptures, 59%), whereas the remaining two (9%) were in women who did not previously undergo any surgery. All of the 13 non-CS ruptures were identified in women with a history of laparoscopic procedures to the uterus. Specifically, 10 (76%) occurred after a previous laparoscopic myomectomy, one (8%) following a hysteroscopic myomectomy, and two (16%) after a laparoscopic wedge resection of cornual ectopic pregnancy. Severe bleeding (blood loss >1500 mL) requiring transfusions was more frequent in women who experienced non-CS compared with CS ruptures (10 *versus* 1 case, respectively, P = 0.024). Patients with a history of endoscopic uterine surgery should be aware of uterine rupture during pregnancy.

## Introduction

Uterine rupture − a serious obstetric emergency and a potentially life-threatening condition to both mother and child–requires immediate surgical intervene for fetal rescue and uterine repair or hysterectomy [1,2]. Although its prevalence varies depending on the population studied and local health service obstetric care, uterine rupture generally occurs in a scarred uterus. In this regard, the presence of a cesarean scar (CS) has traditionally been considered as the main risk factor for uterine rupture, being the underlying etiology in the majority (50−90%) of cases over the past decades. [1,3,4,5,6,7,8]. Notably, the risk of uterine rupture after cesarean delivery is significantly higher (approximately 1%) in trial of labor [9] compared to 2.2 cases per 10,000 elective cesarean deliveries [10,11]. However, recent years have witnessed a significance change in the clinical landscape of uterine rupture [12,13]. Specifically, uterine ruptures at the site of a previous CS are declining as a result of a lower parity and fewer trial of vaginal birth after cesarean delivery (VBAC) [14,15]. In contrast, uterine ruptures attributable to other causes that traumatize the myometrium are increasing and pose significant challenges to obstetric care providers.

Since its introduction, minimally invasive laparoscopic uterine surgery has increasingly been used in women of reproductive age. Although the occurrence of uterine rupture following endoscopic interventions to the uterus has been previously reported [16,17], there has been limited systematic investigation of the potential role played by such procedures in the occurrence of this obstetric emergency [12]. To determine whether changes in the causes of uterine rupture had occurred in recent years and how they could be related to a history of endoscopic uterine surgery, we retrospective retrieved the clinical records of all cases with uterine rupture observed in the delivery room of a Taiwanese tertiary obstetric center over a 15-year period (between July 2001 and July 2016). Complication rates as well as maternal and fetal outcomes were also assessed.

## Materials and methods

All women in this study (S1 Table) were of Taiwanese descent. This study was approved by the local Institutional Review Board. In line with the 2010 National Institutes of Health Consensus Development Conference statement on VBAC, uterine rupture was defined as 1) an anatomic separation of the uterine muscle (with or without symptoms), 2) a disruption or tear of the uterine muscle and the visceral peritoneum, or 3) a separation of the uterine muscle with extension to the bladder or the broad ligament [18]. Women who delivered at less than 14 weeks of gestation, multiple gestation, uterine abnormalities, placenta percreta, induction of labor in intrauterine fetal death, or direct uterine trauma (e.g., fall, motor vehicle accident) were excluded. The following maternal characteristics were collected: demographic variables, medical history, parity, gestational age, and maternal body mass index. We also collected data on potential risk factors for uterine rupture–including a previous CS, induction of labor, mode of delivery, maternal signs and symptoms, surgical approach, and need for blood transfusions. All of the infants were followed up for at least 120 days after birth.

Continuous variables are expressed as medians and ranges and compared with the Mann-Whitney *U* test. Categorical variables were summarized using counts and percentages and analyzed with the Fisher’s exact test. All calculations were performed with IBM SPSS Statistics version 22.0 (IBM Corp, Armonk, NY, USA). Two-tailed P values <0.05 were considered statistically significant.

## Results

### General patient characteristics

**Fig 1:**
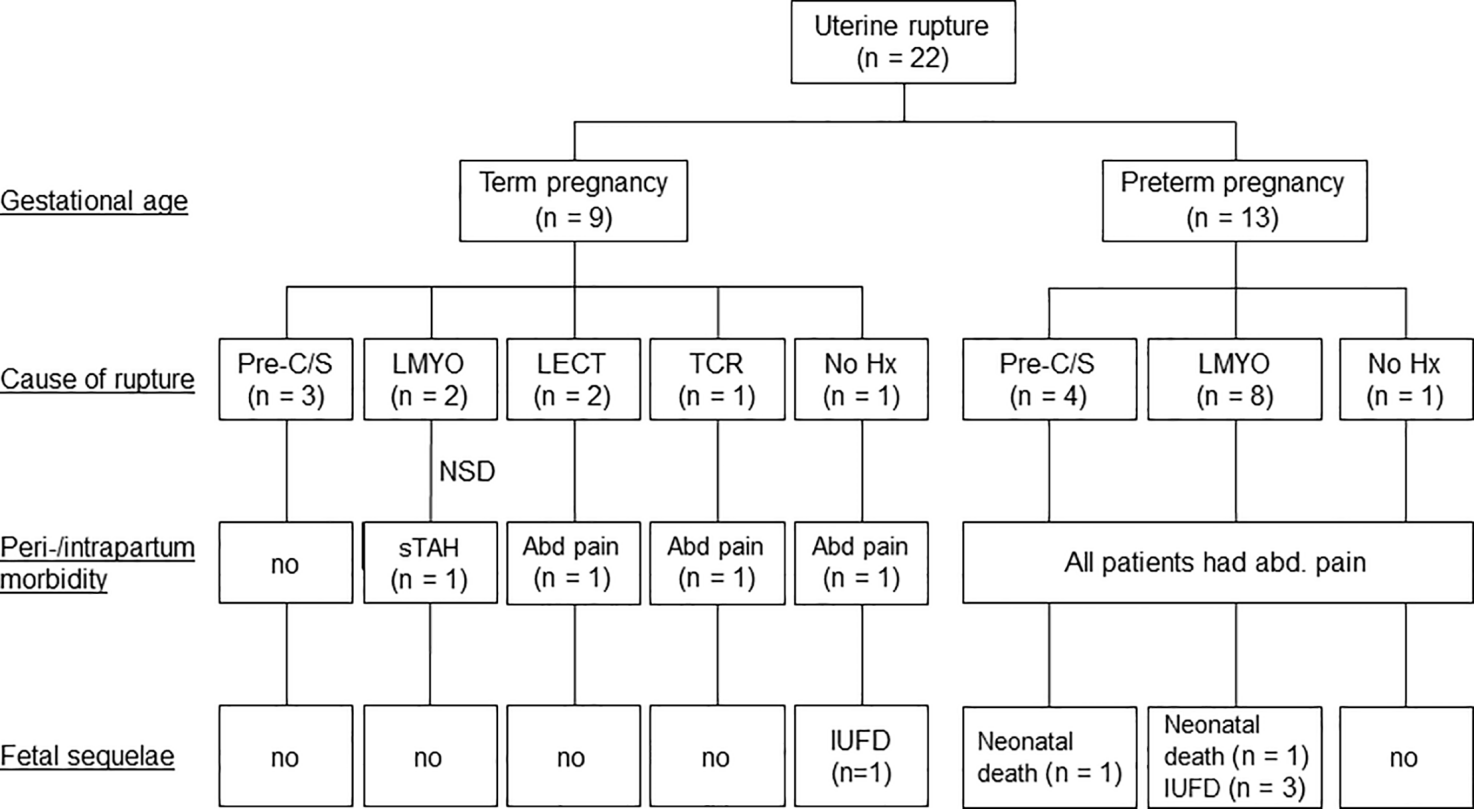
Maternal morbidity and fetal sequelae of women with uterine rupture at different gestational ages. Abbreviations: abd, abdominal; Hx, history; IUFD, intrauterine fetal death; LMYO, laparoscopic myomectomy; LECT, laparoscopic wedge resection of cornual pregnancy, NA, not applicable; Pre-C/S, previous cesarean section; TCR-MYO, trans-cervical resection of myomectomy; sTAH, subtotal hysterectomy.

Over the 15-year study period, a total of 58,076 deliveries occurred. Of them, 37,633 (64.8%) were vaginal and 20,443 (35.2%) were cesarean deliveries. We identified a total of 22 cases of uterine rupture in 20 women (with two of them experiencing two episodes), resulting in an overall uterine rupture rate of 3.8 per 10,000 deliveries. All uterine ruptures occurred in singleton pregnancies. The median age of women who developed uterine rupture was 35 years (range: 20−43 years). Of the 22 uterine ruptures, seven (32%) occurred through a previous cesarean scar (CS ruptures, 32%), 13 through a non-cesarean scar (non-CS ruptures, 59%), and the remaining two (9%) in women who did not previously undergo any surgery. The latter two subgroups were combined for the purpose of analysis. **Table 1** shows the general characteristics of the entire cohort and the two study groups (CS ruptures, n = 7; non-CS ruptures, n = 15). The flow of patients through the study is depicted in **Fig 1**. All of the 13 non-CS ruptures occurred in women with a history of laparoscopic procedures to the uterus. Specifically, 10 (76%) occurred after a previous laparoscopic myomectomy (LMYO), one (8%) following a transcervical resection myomectomy (TCR-MYO), and two (16%) after a laparoscopic wedge resection of cornual ectopic pregnancy (LECT).

**Table 1:** General characteristics, fetal sequelae, and maternal morbidity of women with uterine rupture classified according to the presence or absence of a cesarean scar.

Variable	Entire cohort (n = 22)		CS ruptures (n = 7)		Non-CS ruptures (n = 15)		P
Age (years)	35	(20−43)	34	(20−38)	35	(29−43)	0.501
Body mass index (kg/m^2^)	25	(20−36)	29	(20−36)	25	(22−29)	0.099
Interval from surgery to pregnancy (months)	14.4	(2.5−115.6)	57.3	(6.7−115.6)	7.6	(2.5−65.3)	0.046^a^
Gestational week at uterine rupture	34	(21−40)	33	(26−37)	35	(21−40)	0.569
Preterm delivery	13	(59.1%)	4	(57.1%)	9	(60%)	0.999
Parity (>0)	12	(54.5%)	6	(85.7%)	6	(40%)	0.074
Fetal sequelae							
Neonatal death or IUFD	6	(27.3%)	1	(14.3%)	5	(33.3%)	0.616
Admission to NICU	16	(72.7%)	4	(57.1%)	12	(80%)	0.334
Maternal morbidity							
Shock	4	(19%)	0	(0%)	4	(28.6%)	0.255
Hysterectomy	1	(4.8%)	0	(0%)	1	(7.1%)	0.999
Requirement of blood transfusions	11	(52.4%)	1	(14.3%)	10	(71.4%)	0.024^a^

Data are expressed as medians (ranges) or counts (percentages), as appropriate. Abbreviations: CS, cesarean scar; IUFD, intrauterine fetal death; NICU, neonatal intensive care unit.

^a^Statistically significant difference.

### Comparison of CS and non-CS uterine ruptures

The rupture rates were 6.6 per 10000 (7/9588) for pre-CS and 3.1 per 10000 (15/48488) for non-CS groups. Exact Poisson test was used for this rate ratio comparison. The difference was not statistically significant (P = 0.116). In Taiwan, trial of labor after cesarean accounts for 1.67% of total labor [19]. However, none of the patients in this study had opted VBAC. Of the 22 cases of uterine rupture, 9 (41%) occurred in term pregnancies (i.e., as of 37 weeks of gestation), whereas the remaining 13 (59%) were identified in preterm pregnancies with no option of a trial of labor. The three term ruptures which had a previous CS were not under VBAC (**Fig 1**). The CS and non-CS ruptures did not differ significantly with regard to age, body mass index, gestational week at uterine rupture, and preterm delivery (**Table 1**). However, the time interval from surgery to pregnancy was significantly shorter in the non-CS than in CS ruptures (7.6 months *versus* 57.3 months, respectively, P = 0.046). We also detected a higher trend between groups in terms of parity in the CS ruptures (P = 0.074). Fetal sequelae (i.e., neonatal death, intrauterine fetal death, and admissions to the neonatal intensive care unit) did not differ significantly between the two groups. With regard to maternal morbidity, uterine rupture was successfully repaired in all patients–the only exception being a woman with a history of LMYO who developed a postpartum hemorrhage requiring subtotal hysterectomy (**Fig 1**). However, severe bleeding (blood loss >1500 mL) requiring transfusions was more frequent in women who experienced non-CS compared with CS ruptures (10 *versus* 1 case, respectively, P = 0.024).

### Comparison of uterine ruptures occurring in term and preterm pregnancies

**Table 2** shows the general characteristics, fetal sequelae, and maternal morbidity of women with uterine rupture classified according to the presence of term *versus* preterm pregnancy. Among non-CS ruptures, four cases in term pregnancies (one TCR-MYO, two LECT, and one with a negative history of uterine surgery) presented with abdominal pain and fetal distress. Unfortunately, the woman without a history of previous surgery to the uterus required an emergency section and delivered a full-term newborn with severe hypoxic encephalopathy who died at 6 months of age **(Fig 1)**. All of the 13 uterine ruptures occurring in preterm pregnancies presented with severe abdominal pain and signs of fetal distress. As far as the fetal sequelae were concerned, admissions to the neonatal intensive care unit were significantly more frequent in preterm than in term pregnancies (13 *versus* 3 cases, respectively, P = 0.001; **Table 2)**. A woman who previously underwent LMYO developed an early uterine rupture (at 21 weeks of gestation) accompanied by intrauterine fetal death (IUFD; **Fig 1)**. Among non-CS ruptures, we also observed another neonatal death and two additional cases of IUFD (occurring at the 32, 31, and 24 weeks of gestation, respectively). Finally, a neonatal death occurred at 26 weeks of gestation in a woman with a cesarean scar.

**Table 2:** General characteristics, fetal sequelae, and maternal morbidity of women with uterine rupture classified according to term *versus* preterm pregnancy.

Variable	Term pregnancy (n = 9)		Preterm pregnancy (n = 13)		P
Age (years)	33	(20−43)	36	(29−39)	0.565
Body mass index (kg/m^2^)	26	(23−36)	25	(20−31)	0.361
Interval from surgery to pregnancy (months)	19.6	(2.5−115.6)	14.4	(4.6−105)	0.792
Parity (>0)	6	(66.7%)	6	(46.2%)	0.415
Fetal sequelae					
Neonatal death or IUFD	1	(11.1%)	5	(38.5%)	0.333
Admission to NICU	3	(33.3%)	13	(100%)	0.001^a^
Maternal morbidity					
Shock	2	(22.2%)	2	(16.7%)	0.999
Hysterectomy	1	(11.1%)	0	(0%)	0.429
Requirement of blood transfusions	4	(44.4%)	7	(58.3%)	0.670

Data are expressed as medians (ranges) or counts (percentages), as appropriate. Abbreviations: IUFD, intrauterine fetal death; NICU, neonatal intensive care unit. ^a^Statistically significant difference.

## Discussion

The main findings of the current study were as follows: 1) patients with a history of endoscopic uterine surgery should be aware of uterine rupture during pregnancy; 2) the time interval from surgery to pregnancy was significantly shorter in the non-CS than in CS ruptures; 3) severe bleeding (blood loss >1500 mL) requiring transfusions was more frequent in women who experienced non-CS compared with CS ruptures; and 4) when uterine rupture occurred, admissions to the neonatal intensive care unit were significantly more frequent in preterm than in term pregnancies.

The overall incidence of uterine rupture observed in the current 15-year study (3.8 per 10,000 pregnancies) was higher than that previously reported in the literature for women with a CS (2.2 per 10,000 pregnancies) [9,10]. This observation suggests that the burden of uterine rupture has recently increased and its underlying causes have changed considerably from previous section. In the current study, the frequency of non-CS ruptures (59%) was higher than that of the traditional CS ruptures (32%), with all of them occurred in women with a history of endoscopic procedures to the uterus. Although case reports of uterine rupture following endoscopic uterine surgery have been published [16,17,20,21,22,23,24,25,26], other authors suggested that laparoscopic myomectomy can be safely performed in women of reproductive age who want to become pregnant [6,7,8,27]. Notwithstanding that further research is needed to ascertain the exact impact of different endoscopy techniques, our data suggest that the risk of uterine rupture associated with the presence of a uterine scar following laparoscopic surgery may be even higher than that conferred by a cesarean scar. Taken together, our findings point toward a changing landscape for uterine rupture − with the risk of CS-related rupture currently decreasing (as a result of a lower parity and fewer option for VBAC) and the rates of non-CS ruptures showing a raising pattern (the latter phenomenon being a consequence of the increasing use of uterine endoscopic procedures).

Another important finding of the current study is that the time interval from surgery to pregnancy was significantly shorter in the non-CS than in CS ruptures. Because healing of a uterine scar occurs over time, we hypothesize that the time interval from surgery to pregnancy is an important factor influencing the risk of uterine rupture [12]. In light of our findings, we believe that women who undergo endoscopic uterine interventions should be counseled about the optimal timing of pregnancy after surgery to minimize the likelihood of uterine rupture. Intriguingly, we also observed that severe bleeding (blood loss >1500 mL) requiring transfusions was more frequent in women who experienced non-CS compared with CS ruptures. In this scenario, screening tests before conception (e.g., assessment of the uterine cavity and the myometrium by imaging and/or hysteroscopy) as well as a thorough investigation of the uterine wall and placentation during the first trimester of pregnancy should be considered in women who had previously undergone endoscopic surgery to the uterus. Finally, the present study indicates that admissions to the neonatal intensive care unit were significantly more frequent when uterine rupture occurred in preterm than in term pregnancies − suggesting that this condition poses special challenges in terms of fetal outcomes.

Despite some limitations (e.g., small sample size and exclusive inclusion of women who delivered after 14 weeks of gestation), our data are provocative and offer several areas for further research. For example, the exact mechanisms by which laparoscopic interventions to the uterus can increase the risk of uterine rupture deserve further scrutiny, although they are probably related to structural traumas of the myometrium. Another potential area for future investigation involves the potential reduction of non-CS uterine ruptures by increasing the time interval from endoscopy to pregnancy, (especially in nulligravidae having childbearing intentions). In addition, larger case studies and case-control analyses might provide some guidelines for trial of labor following uterine surgery. This knowledge would be helpful to counseling women undergoing endoscopic uterine surgery, on the ideal timing of pregnancy after endoscopic uterine interventions, and awareness of obstetric complications to optimize subsequent pregnancy outcomes.

## Conclusions

Patients with a history of endoscopic uterine surgery should be aware of uterine rupture during pregnancy. Given the high burden of fetal morbidity and mortality associated with preterm uterine rupture, continued vigilance over this condition is paramount.

## Supporting information

S1 Table “ut rupture_PONE”. (XLS)
